# Combined heat and water stress leads to local xylem failure and tissue damage in pyrethrum flowers

**DOI:** 10.1093/plphys/kiad349

**Published:** 2023-06-16

**Authors:** Madeline R Carins-Murphy, Hervé Cochard, Ross M Deans, Alistair J Gracie, Timothy J Brodribb

**Affiliations:** School of Natural Sciences, Discipline of Biological Sciences, University of Tasmania, Hobart, Tasmania 7001, Australia; INRAE, PIAF, Université Clermont-Auvergne, Clermont-Ferrand 63000, France; Department of Viticulture & Enology, University of California, Davis, California 95616, USA; Tasmanian Institute of Agriculture, University of Tasmania, Hobart, Tasmania 7001, Australia; School of Natural Sciences, Discipline of Biological Sciences, University of Tasmania, Hobart, Tasmania 7001, Australia

## Abstract

Flowers are critical for angiosperm reproduction and the production of food, fiber, and pharmaceuticals, yet for unknown reasons, they appear particularly sensitive to combined heat and drought stress. A possible explanation for this may be the co-occurrence of leaky cuticles in flower petals and a vascular system that has a low capacity to supply water and is prone to failure under water stress. These characteristics may render reproductive structures more susceptible than leaves to runaway cavitation—an uncontrolled feedback cycle between rising water stress and declining water transport efficiency that can rapidly lead to lethal tissue desiccation. We provide modeling and empirical evidence to demonstrate that flower damage in the perennial crop pyrethrum (*Tanacetum cinerariifolium*), in the form of irreversible desiccation, corresponds with runaway cavitation in the flowering stem after a combination of heat and water stress. We show that tissue damage is linked to greater evaporative demand during high temperatures rather than direct thermal stress. High floral transpiration dramatically reduced the soil water deficit at which runaway cavitation was triggered in pyrethrum flowering stems. Identifying runaway cavitation as a mechanism leading to heat damage and reproductive losses in pyrethrum provides different avenues for process-based modeling to understand the impact of climate change on cultivated and natural plant systems. This framework allows future investigation of the relative susceptibility of diverse plant species to reproductive failure under hot and dry conditions.

## Teaser

Transpiration-induced runaway cavitation in flowering stems of the perennial crop pyrethrum (*Tanacetum cinerariifolium*) drives flower damage during hot and dry conditions.

## Introduction

Global ecosystems and the production of food, fiber, and pharmaceuticals rely on healthy flowers. Flowering plants account for nearly 90% of land plant biodiversity (Crepet and Niklas 2009) and up to 90% of the human diet (Şerban et al. 2008) with most staple foods derived from seeds (Cassman 1999). They also include the ubiquitous fiber crop cotton (*Gossypium hirsutum*) and the opium poppy (*Papaver somniferum*). However, flowers are likely to be particularly impacted by the changing global climate (Patiño and Grace 2002; Hedhly et al. 2009; Borghi et al. 2019; Roddy 2019). Rainfall is predicted to become increasingly variable in the coming decades and heat events more frequent and severe in most regions (IPCC 2014). As a result, the 2 climatic stresses of drought and heat will converge more often. Many plants shed their flowers during heat and drought events in isolation, causing yield losses in crops such as legumes, grapevine (*Vitis vinifera*), and pyrethrum (*Tanacetum cinerariifolium*) (Warrag and Hall 1984; Li et al. 1991; Guilioni et al. 1997; Warner and Erwin 2005; Fang et al. 2010; Greer and Weston 2010; Guo et al. 2013; Greyvenstein et al. 2014; Suraweera et al. 2020). However, the co-occurrence of drought and hot weather during reproductive growth has an especially severe impact on yield (Cohen et al. 2021). Although progress has been made in identifying flower metabolic responses to abiotic stress (Borghi et al. 2019) and the phytohormone/peptide signaling pathways that regulate plant organ shedding during stress (Reichardt et al. 2020), the upstream processes that initiate flower damage, senescence, and abscission during hot and dry conditions are poorly understood. This limits our capacity to accurately forecast the likelihood of injury to crop and native plant reproduction, and subsequent impacts on ecosystem function and food security, as the global climate changes.

If soil water is plentiful, water lost through open stomata can maintain leaves at viable temperatures during hot conditions via evaporative cooling (Beerling et al. 2001). Water loss across the floral cuticle may protect gametes from thermal injury in a similar way. Floral organs tend to have no or fewer stomata (Lipayeva 1989; Feild et al. 2009; Lambrecht et al. 2011; Zhang et al. 2018; Lin et al. 2020) and a greater conductance to water vapor than leaves once stomata are closed (Nobel 1977; Buschhaus et al. 2014; Cheng et al. 2019; Bourbia et al. 2020; Cheng et al. 2021), with some exceptions (Whiley et al. 1988). This indicates that in many species, floral cuticles are a weaker barrier to water loss and/or that floral stomata are leakier compared with those of leaves. Furthermore, field studies show that evaporation from the perianth significantly cools the gynoecium of some tropical species during the day (Patiño and Grace 2002). Thus, producing flowers with relatively high residual conductance to water vapor (*g*_res_) may be an adaptation to cool gametes in hot conditions without a large investment in stomata or the regulation of their aperture. Recent work observed “reproductive segmentation” in the Mediterranean daisy pyrethrum, whereby the flower (a term used here and throughout the rest of the text to encompass the entire inflorescence or capitulum of pyrethrum) has higher *g*_res_ than the leaf and is shed first during drought before any leaf damage (Bourbia et al. 2020). It was hypothesized that floral water loss could trigger xylem dysfunction in the flowering stem, hydraulically isolating flowers from the rest of the plant. Being perennial, this species can then defer reproduction until the next year. When heat and drought are experienced in isolation, unregulated floral water loss may, therefore, function to keep gametes at a viable temperature or defer reproduction until soil water is available, respectively. It may similarly underpin the negative effect of combined heat and drought on flower retention.

The disruption of water transport during localized tissue dehydration (Tyree and Sperry 1988) has been explored as a mechanistic explanation for sudden damage to vegetative plant organs when hot weather co-occurs with drought (Cochard 2019; Brodribb et al. 2020). During normal conditions, water is “pulled” from the soil into plant roots and up through the internal water transport system (the xylem) to replenish water lost from leaves as transpiration. This permits gas exchange for photosynthesis but exposes plants to the risk of catastrophic failure of water supply because water transport occurs under a tension that increases as soil dries and/or transpiration increases. Under extreme tension, air blockages (embolisms) form in the xylem in a process called xylem cavitation, reducing water transport capacity (Tyree and Sperry 1989). Stomatal closure slows this process by reducing water loss and hence the rate of dehydration, allowing a degree of homeostasis in plant water content. However, some residual transpiration always continues through the cuticle and closed stomata creating an uncontrolled evaporative pathway with the potential to induce dehydration damage to the vascular system and downstream tissues. High temperatures intensify this water loss by increasing the evaporative driving force (vapor pressure deficit [VPD]) and, in some species, triggering a steep increase in residual conductance to water vapor (Duursma et al. 2018). If residual transpiration causes water potential to fall sufficiently to initiate cavitation, or some embolisms are already present due to existing water stress, then a feedback loop can develop between declining xylem water potential and water transport capacity due to increasing xylem cavitation (so called runaway cavitation), culminating in complete blockage of water transport, tissue desiccation, and death (Tyree and Sperry 1988; Brodribb et al. 2021; Tonet et al. 2023).

It remains to be tested whether this catastrophic feedback loop occurs in floral tissues, but evidence from pyrethrum suggests that floral tissues of this species could be predisposed to runaway cavitation, especially when evaporative demands are high during hot conditions. Pyrethrum flowers are a greater source of residual transpiration, and pyrethrum flowering stems have a lower capacity to replenish water lost from transpiration and have xylem more vulnerable to cavitation under water stress, than leaves (Bourbia et al. 2020). We hypothesize that the combination of these traits will cause pyrethrum flowering stems to be susceptible to runaway cavitation during heat and that water stress prior to heat will position them closer to this tipping point. We further hypothesize that the severing of the water supply, caused by runaway cavitation, will rapidly and irreversibly desiccate the flowers. To explore these hypotheses, we use a mechanistic hydraulic model to simulate the impact of a short-term heat event on leaves and flowers at different levels of water stress to determine the relative susceptibility of flowering stems to hydraulic damage when soil–plant–atmosphere interactions are considered. We then use an image-based technique (Brodribb, Bienaimé, et al. 2016; Brodribb, Skelton, et al. 2016) to monitor cavitation in individual flowering stems in situ during experimental heat exposure of potted plants previously subjected to varying degrees of water stress to find direct evidence linking cavitation to flower mortality. Plants receiving no or mild water stress test the alternative hypothesis that thermal stress damages flowers directly, without associated dehydration. Our study aims to provide a framework to predict the susceptibility of diverse plant species to reproductive failure under hot and dry conditions using key floral traits.

## Results

### Residual transpiration and conductance to water vapor

**Figure 1.**
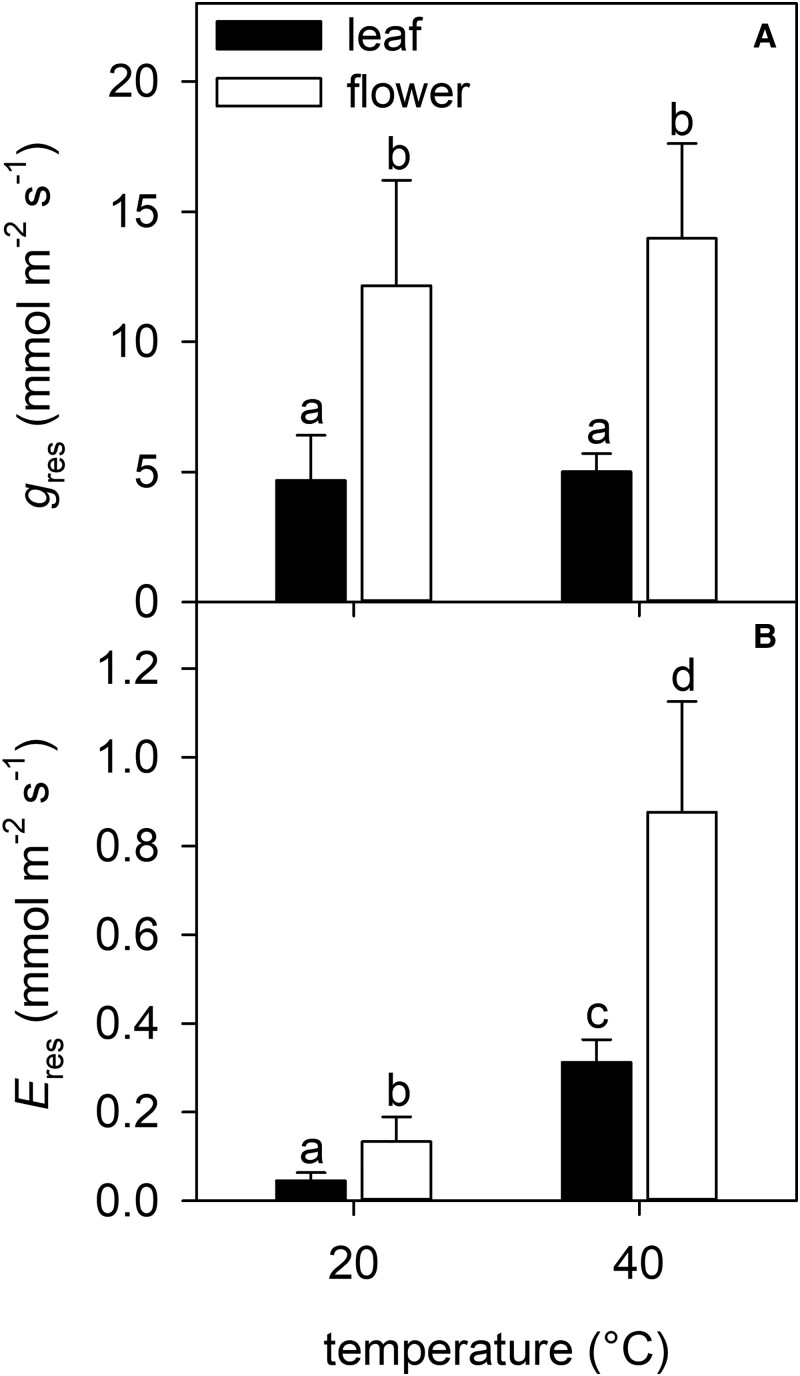
Pyrethrum flowers have greater conductance to water vapor after stomatal closure than leaves at both 20 and 40°C. Residual conductance to water vapor (*g*_res_) **A)**, and transpiration (*E*_res_) **B)** at 20 and 40°C of leaves and flowers exposed to water stress predicted to close stomata. Columns are means + Se (*n* = 3 individuals). Different letters indicate significant differences among means: a-b and c-d (*P* < 0.05), a-c and b-d (*P* < 0.001) (*g*_res_: organ *F*_1,8_ = 9.922, *P* < 0.05; temperature *F*_1,8_ = 0.401, *P* > 0.05; organ:temperature *F*_1,8_ = 0.001, *P* > 0.05) (*E*_res_: organ *F*_1,8_ = 8.954, *P* < 0.05; temperature *F*_1,8_ = 29.646, *P* < 0.001; organ:temperature *F*_1,8_ = 1.685, *P* > 0.05).

*E*_res_ was >2-fold greater in pyrethrum flowers than that in leaves at both temperatures (20°C: 0.13 ± 0.06 versus 0.05 ± 0.02 mmol m^−2^ s^−1^; 40°C: 0.88 ± 0.25 versus 0.31 ± 0.05 mmol m^−2^ s^−1^) (Fig. 1). Increasing the ambient temperature from 20 to 40°C resulted in a 6-fold increase in *E*_res_ of both flowers and leaves (organ *F*_1,8_ = 8.954, *P* < 0.05; temperature *F*_1,8_ = 29.646, *P* < 0.001; organ:temperature *F*_1,8_ = 1.685, *P* > 0.05) (*n* = 3 individuals). Likewise, pyrethrum flowers had a >2-fold greater *g*_res_ than that of leaves at both temperatures (20°C: 12.15 ± 4.05 versus 4.68 ± 1.73 mmol m^−2^ s^−1^; 40°C: 13.98 ± 3.64 versus 5.01 ± 0.7 mmol m^−2^ s^−1^) (Fig. 1). Increasing the ambient temperature from 20 to 40°C had no significant effect on *g*_res_ of either organ (organ *F*_1,8_ = 9.922, *P* < 0.05; temperature *F*_1,8_ = 0.401, *P* > 0.05; organ:temperature *F*_1,8_ = 0.001, *P* > 0.05).

### Theoretical susceptibility of flowers versus leaves to runaway cavitation during heat

Based on the mathematically derived threshold of xylem damage predicted to trigger runaway cavitation (equation (3)), the magnitude of xylem damage (percentage loss of hydraulic conductance [PLC]) predicted to trigger runaway cavitation was lower in flowers than that in leaves, particularly at high temperature (40°C) (Table 1). Uncontrolled runaway cavitation in the leaf xylem was only predicted to occur when the vast majority of xylem conductance was already damaged by cavitation (98.8% and 96.4% loss of hydraulic conductance at 20 and 40°C, respectively). In flowers, runaway cavitation was predicted to occur at much more modest levels of xylem damage (75.6% and 47% loss of hydraulic conductance at 20 and 40°C, respectively).

**Table 1.** Theoretical loss of hydraulic conductance predicted to trigger runaway cavitation (PLC_runaway_) in pyrethrum flowers and leaves at 20 and 40°C

		Flower		Leaf	
Equation prediction	Units	20°C	40°C	20°C	40°C
Theoretical PLC_runaway_	%	75.6	47.0	98.8	96.4

We derived analytical equations to describe the PLC_runaway_ for an organ-specific rate of residual water loss (*E*_res_). At this critical point, the flow of water through the plant organ (*J*_max_) is equal to *E*_res_. If organ water potential decreases further, the resulting cavitation will reduce *J*_max_. Then, *J*_max_ will be less than *E*_res_ triggering a runaway cavitation feedback cycle. See Tonet et al. (2023) for details

### Simulated heat wave disables water transport in the flowers but not leaves of plants under mild water stress

**Figure 2.**
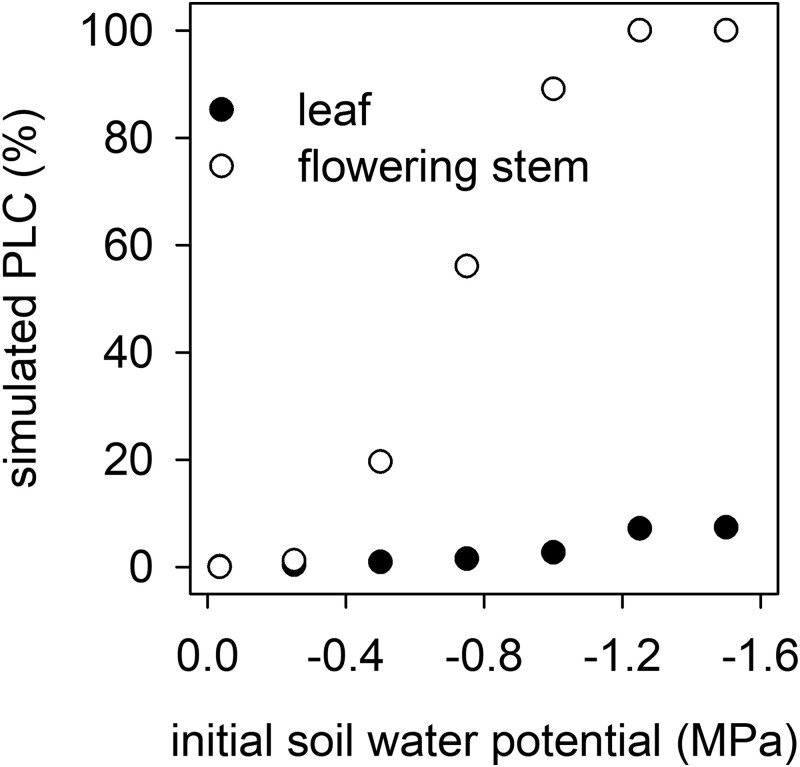
Simulated PLC incurred by pyrethrum leaves and flowering stems during a 3-h 40°C heat event as a function of the soil water potential at onset of heat. Simulations predict that flowering stems undergo complete hydraulic failure during a short-term heat event when soil water potential is less than −1.25 MPa.

**Figure 3.**
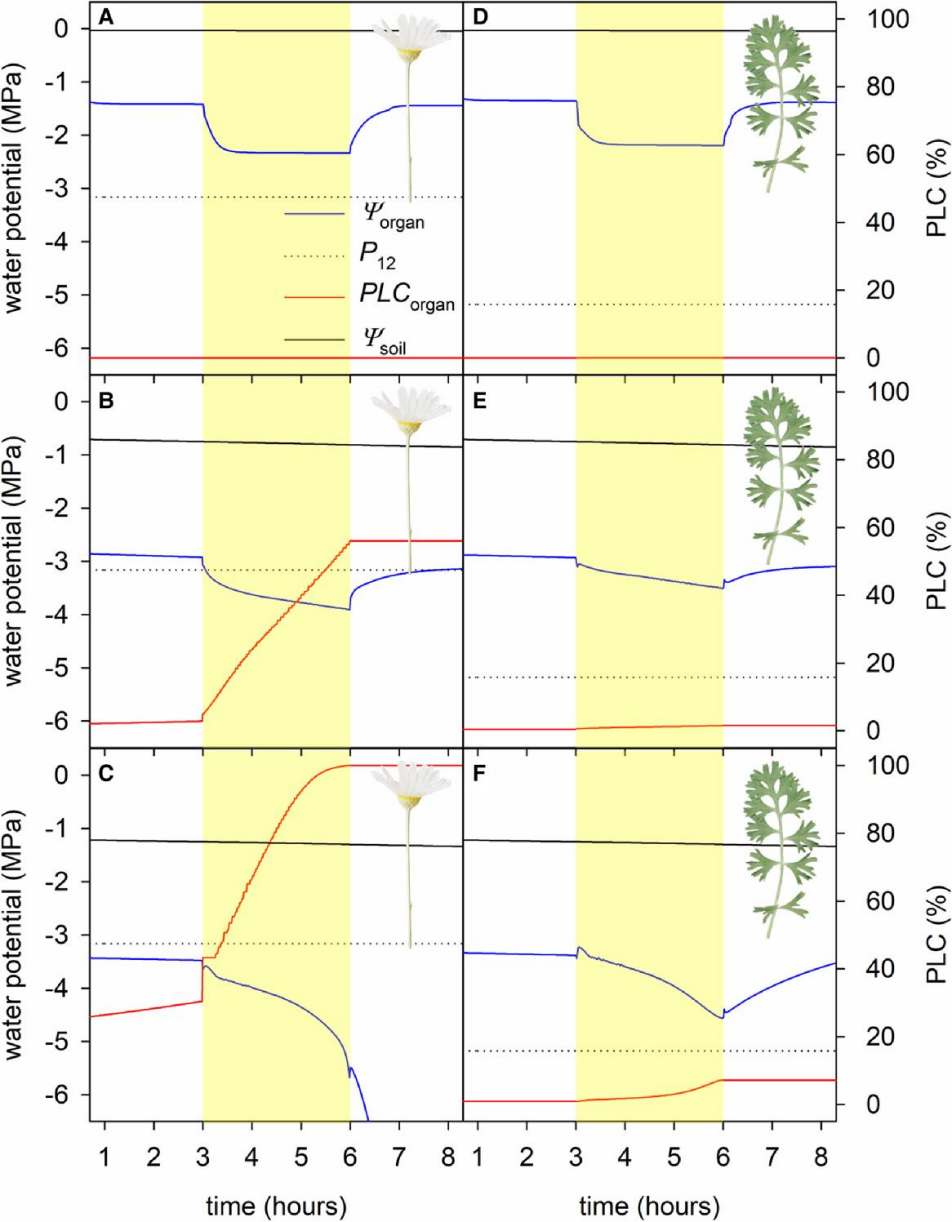
Representative simulations with SurEau predict the effect of a heat wave (ambient = 25°C, heat wave = 40°C) on pyrethrum leaves and flowers exposed to different levels of initial drought stress. The results of 3 simulations are shown using an initial soil water potential (*ψ*_soil_) of either 0 **A** and **D)**, −0.75 **B** and **E)**, or −1.25 MPa **C** and **F)**. Solid lines show changes in organ water potential (*ψ*_organ_; blue), percentage loss of conductance (*PLC*_organ_; red), and soil water potential (*ψ*_soil_; black). Yellow shading indicates the heat wave timing. Dotted lines show the water potential of incipient (12%) cavitation in the flowering stem and leaf xylem (*P*_12_) measured in a previous study (Bourbia et al. 2020).

Simulations of water flow and cavitation dynamics using the SurEau soil–plant–atmosphere hydraulic model predicted that pyrethrum flowers would undergo complete hydraulic failure (i.e. 100% PLC) during the 3-h 40°C heat treatment when initial soil water potential was −1.25 MPa (Figs. 2 and 3, A to C). This initial soil water potential corresponded to a 30% loss of flower hydraulic conductance, close to the calculated percentage loss of conductance value for runaway cavitation (PLC_runaway_) using equation (3) of 47%. The prediction of hydraulic failure in this case was due to dehydration and cavitation within the plant and not due to changes in soil water content (decline in soil water potential during heat across all simulations was predicted to be minimal [mean ± Se: −0.046 ± 0.007 MPa]). Furthermore, leaves, with their lower vulnerability to cavitation, were predicted to incur only minor hydraulic damage (<7.5% loss of hydraulic conductance) during simulated heat events (Figs. 2 and 3, D to F).

### Short-term experimental heat stress superimposed on mild water stress triggers rapid hydraulic failure and death in mature flowers but has little effect on leaves

**Figure 4.**
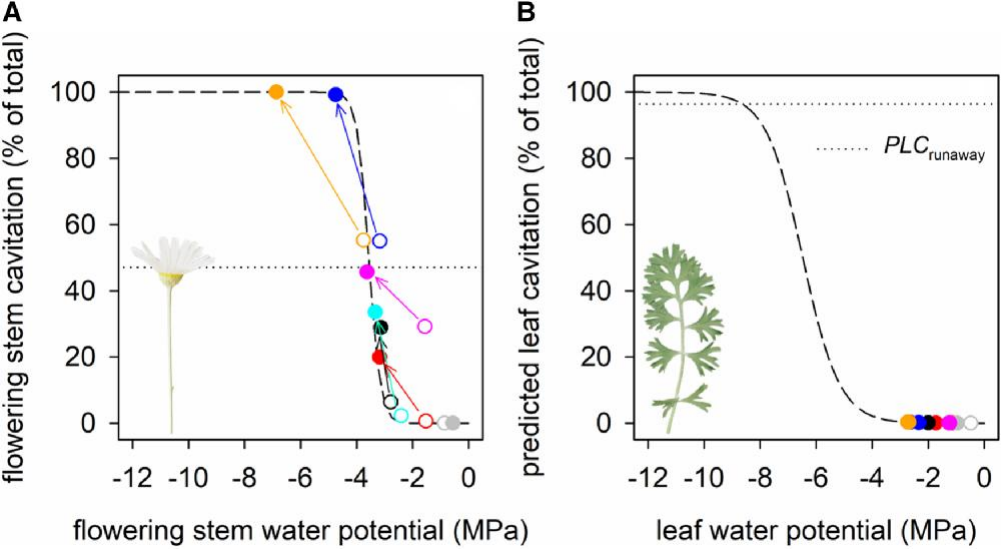
Short-term experimental heat stress triggers a greater decline in water potential and increase in cavitation in pyrethrum flowering stems than in leaves. Relationship between organ water potential and cavitation (% of total) in the flowering stem **A)** and leaf **B)** before (open symbols) and after (filled symbols) heat exposure. Symbols of the same color connected with an arrow in the same panel show values from the same organ, and symbols of the same color in different panels show values from different organs from the same individual. Gray symbols show the well-watered individual. All other symbol colors show individuals subjected to water stress before heat exposure. Dashed lines describe the fitted sigmoidal equation for the relationship between water potential and cavitation measured in a previous study for flowering stems [slope parameter (*a*) = 4.89 and the water potential at which 50% of vessels are cavitated (*P*_50_) = −3.57] and for leaves (*a* = 1.53 and *P*_50_ = −6.48) (Bourbia et al. 2020). Dotted horizontal lines show the theoretical loss of conductance that triggers runaway cavitation (PLC_runaway_) at 40°C (flower = 47%; leaf = 96.4%).

Theoretical predictions of flower hydraulic failure during heat combined with mild water stress were supported empirically by experimental observations. In all cases, except for the well-watered plant (gray symbols), flowering stem xylem was partially embolized (to a maximum of 55%) due to water stress imposed prior to the heat treatment (open symbols; Fig. 4A). Exposure to heat (40°C) differentially increased flowering stem water stress (i.e. reduced flowering stem water potential) relative to leaves in all cases except the well-watered plant, with a mean ± Se decline (excluding the well-watered plant) of 1.6 ± 0.4 MPa in flowering stems and 0.06 ± 0.1 MPa in leaves (*P* < 0.05) (Fig. 4). Increased flowering stem water stress was associated with an increase in flowering stem xylem cavitation measured in vivo in all cases except for the well-watered plant. The water potential that was expected to produce incipient xylem cavitation in the flowering stem xylem (*P*_12_ = −3.2 MPa; Bourbia et al. 2020) was never approached in the well-watered control plant. The severity of hydraulic injury after heat exposure corresponded to the water stress imposed on plants prior to heat exposure. Thus, a rapid transition to complete hydraulic failure during heat occurred in the 2 most water stressed flowering stems that had the greatest percentage of cavitated vessels prior to heat exposure (Fig. 4A). These were the only 2 flowering stems where the loss of hydraulic conductance exceeded the theoretical tipping point for runaway cavitation (i.e. 47%). Furthermore, the rate of cavitation propagation in flowering stems during heat increased with existing cavitation load before heat (Supplemental Fig. S1). Plant water potential remained stable despite a transient increase in evaporation during the experimental heat treatment (*P* > 0.05) (Supplemental Fig. S2).

**Figure 5.**
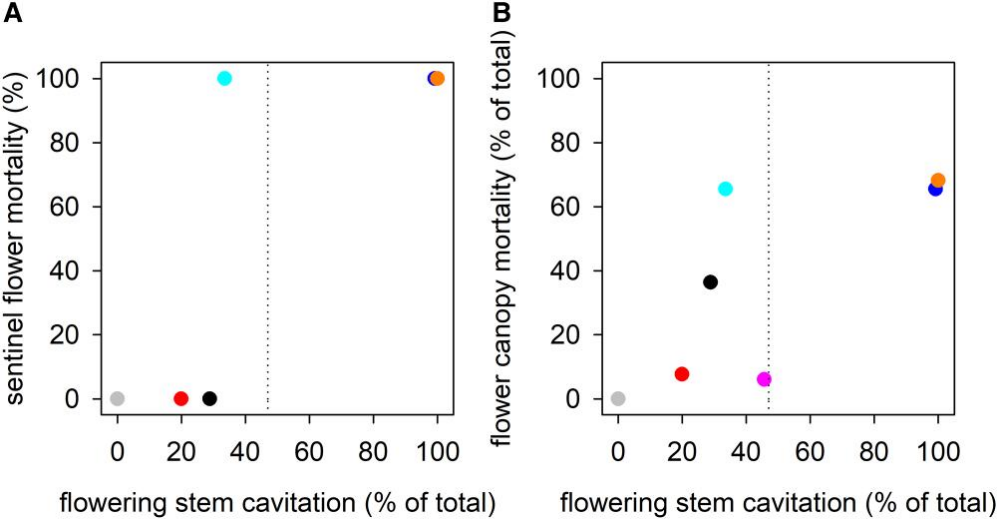
Flower damage followed short-term heat stress when flowering stems upstream of sentinel flowers approached or surpassed the theoretical loss of conductance that triggers runaway cavitation at 40°C. Mortality of sentinel flowers **A)** and the flower canopy (% of total mature flowers per plant) **B)** varied with cavitation in flowering stems upstream of sentinel flowers following experimental heat stress. Symbols of the same color represent values from the same individual. Dotted vertical lines show the theoretical loss of conductance that triggers runaway cavitation at 40°C (47%).

Sentinel flower mortality varied with upstream flowering stem cavitation following heat exposure (Fig. 5A). Three out of the 4 sentinel flowers supported by flowering stems, which lost less hydraulic conductance following heat than the theoretical tipping point for runaway cavitation (i.e. PLC was <47%) survived (Fig. 5A). However, a fourth flower died following heat exposure when only 34% of flowering stem vessels became nonfunctional. Both sentinel flowers supported by flowering stems that underwent hydraulic failure during heat exposure died. Flower canopy mortality, expressed as a percentage of total flowers in the canopy, also varied with flowering stem cavitation upstream of sentinel flowers following heat exposure (Fig. 5B). However, there was variation in floral mortality within the canopy. A large proportion of the flower canopy died (∼67%) when losses in hydraulic conductance in the flowering stems of sentinel flowers approached or exceeded the theoretical tipping point for runaway cavitation, except in 1 case where canopy mortality remained low. Mature flowers also tended to be more sensitive to heat than developing flower buds (Supplemental Fig. S3). Bud death was only observed in 3 plants. Two of these had the greatest flower mortality, and in these cases, far fewer buds died than mature flowers (25% versus 66%, respectively, and 20% versus 68%, respectively). In a third plant with bud death, both bud and mature flower mortality was low (18% versus 6%, respectively). No mature flowers died when water stress expected to cause cavitation in up to 83% of flowering stem xylem area was imposed on additional plants kept at mild temperatures (day and night temperatures of ∼21 and 18°C, respectively) (Supplemental Table S1). Flower mortality was only observed when plants in mild temperatures were subjected to water stress expected to cause cavitation in ∼99% of flowering stem xylem area.

In comparison, leaves of plants exposed to heat never approached the water potential known to induce air entry into the leaf xylem (*P*_12_ = −5.2 MPa) (Bourbia et al. 2020), with postheat leaf water potential ranging from −0.98 to −2.7 MPa (Fig. 4B). Minimal leaf death was observed postheat, and heat had no significant effect on the chlorophyll fluorescence parameter Fv/Fm in leaves, with a mean ± Se Fv/Fm of 0.83 ± 0.003 and 0.81 ± 0.015 before and after heat exposure, respectively (*P* > 0.05).

## Discussion

Despite the particular sensitivity of flowers and crop yields to combined heat and water stress (Cohen et al. 2021) and the threat this poses to food security and native ecosystem function under the changing global climate (IPCC 2014), the primary mechanisms initiating flower damage during hot and dry conditions remain unknown. Here, we present theoretical and empirical evidence supporting the conclusion that extreme evaporative conditions experienced during heat can trigger the process of runaway cavitation in the xylem water supply to pyrethrum flowers under mild drought stress, causing flower mortality. Both analytically and numerically derived models agreed that hot conditions (40°C) greatly increase the likelihood of runaway cavitation in flowering stems but not leaves. Observations of localized water status and cavitation propagation in the flowering stems of plants subjected to different levels of water stress and exposed to heat in situ provided direct empirical support for our theoretical predictions. Together, this evidence identifies transpiration-induced runaway xylem cavitation as the most likely cause of flower mortality in pyrethrum during combined heat and drought stress. Thus, we show that certain traits (high residual floral water loss, low capacity to transport water through floral tissue, and high vulnerability of flowering stem xylem to cavitation) make pyrethrum flowering stems more susceptible to runaway cavitation during heat than leaves and that increased floral transpiration during high temperatures decreases the soil water deficit at which runaway cavitation is triggered. Even a mild decline in soil water content positions flowering stems closer to this lethal tipping point.

We capitalized on recent technological advances that permit cavitation detection in vivo at the high temporal resolution required to link cavitation propagation with short-term heat wave exposure. This allowed us to directly link the application of a heat treatment with paired measurements of flowering stem water potential and cavitation accumulation (i.e. cavitation during heat was always associated with a decline in flowering stem water potential). The well-watered plant demonstrated that cavitation and flower damage was not triggered by heat alone. This supports the conclusion that localized dehydration increased the likelihood of cavitation rather than direct thermal stress. Previous studies have used the optical technique to measure the timing and dynamics of cavitation propagation in a variety of plant tissues, with the resulting vulnerability to cavitation metrics closely corresponding to those produced using other X-ray and hydraulic techniques (Brodribb et al. 2017; Skelton et al. 2017; Gauthey et al. 2020). Our study monitored cavitation in floral tissue in vivo during variable evaporative conditions. Thus, our findings provide important information about the way in which cavitation propagates in plants under field conditions, providing a mechanistic connection between abiotic stress and the well-documented negative effect of heat on flower retention (Warrag and Hall 1984; Li et al. 1991; Warner and Erwin 2005; Greer and Weston 2010; Greyvenstein et al. 2014) and combined heat/drought effect on crop yields (Cohen et al. 2021).

Close agreement between the predicted and observed thresholds leading to runaway cavitation during heat stress provides strong support for the argument that heat can trigger a runaway cavitation sequence in flowering stems once a subset of vessels is rendered nonfunctional. This process also offers a mechanistic explanation for the lethal desiccation of plant tissues in general and thus may underlie the common association between tree die-off events and the co-occurrence of water and high temperature stress (Mitchell et al. 2014). Our data demonstrate that the susceptibility of plant tissues to runaway cavitation can be reliably predicted using knowledge of rather basic hydraulic and evaporative parameters. This provides a framework for future studies to identify the plant species and particular organs within plants that are most threatened by the co-occurrence of heat waves and drought. Although our analyses indicate that runaway cavitation may only be triggered in leaves at very high temperatures and/or following damage to a large proportion of the water transport capacity, equation (3) only considers a simplified scenario in which the leaf water supply begins at the petiole. Thus, the substantial resistances to water flow present upstream of the petiole between the soil and the leaf (Bourbia et al. 2021) might lead to the prediction of an earlier cavitation feedback cycle.

Failure to recover petal turgor after heat stress and rewatering was assumed to indicate flower mortality because it was previously linked with a lack of floral disc expansion in drought stressed pyrethrum plants (Bourbia et al. 2020). This assumption is supported by other work in which pyrethrum flower senescence was observed following a longer but less severe heat treatment (Suraweera et al. 2020). However, mortality of the sentinel flowers monitored for cavitation in our study varied somewhat from the mortality of the total flower canopy, indicating that within plants, flowers experienced different levels of stress or had different sensitivities to heat. This may be related to variation in flower developmental stage, hydraulic characteristics of the supporting stem, or evaporative surface area. Variation between flower bud and mature flower mortality further supports this idea. Resolving the reasons for this variation would improve the accuracy of calculated trade-offs between irrigation input and yield reductions and predictions of flower retention sensitivity (and thus, reproductive output) to temperature fluctuations in wild populations.

The results of our investigation using pyrethrum raise the possibility that evaporation-induced runaway cavitation may also trigger the flower shedding observed in other species following heat exposure (with or without preexisting water stress). Although some previous work has documented declines in flower water potential during the day when temperatures are high (Tsukaguchi et al. 2003), most studies investigating flower susceptibility to heat have not measured floral organ water status (Warrag and Hall 1984; Li et al. 1991; Guilioni et al. 1997; Warner and Erwin 2005; Greer and Weston 2010; Greyvenstein et al. 2014). This raises the question as to whether flower shedding in these species is due to direct thermal damage or, as found here, indirect heat damage from dehydration. Previous work proposed that the preferential shedding of pyrethrum flowers before vegetative organs during drought, with flowering stem cavitation functioning like a hydraulic fuse, may be an adaptation that protects the rest of the plant from the negative effects of excessive floral water loss (Bourbia et al. 2020). This is expected to increase the likelihood of ongoing survival in perennial species like pyrethrum where reproduction can be deferred to the following season and so may be a common adaptation in perennial plants from dry environments. We found that this “floral segmentation” in which cavitation in the flowering stem is induced at a higher (less negative) water potential than in leaves is further exacerbated during heat. Not only did flowering stems have lower tolerance to losses in hydraulic capacity, but also the minimum water potential reached by the flowering stem was also lower than that of leaves. Thus, even if the xylem of flowering stems and leaves had similar vulnerability to cavitation, flowering stems would be damaged first during heat because they experience more negative water potentials than leaves (due to a leakier flower cuticle and low capacity to transport water) and undergo runaway cavitation when less hydraulic capacity is lost. Although declines in leaf water potential predicted by SurEau under high evaporative conditions were larger than observed in experimental plants, in both cases, leaf water potential remained well above cavitation thresholds. Prioritizing vegetative tissues may be problematic for annual species, however, in which reproduction and plant senescence occurs within 1 year. There is evidence that reproductive tissues in annual plants are more resistant to cavitation than vegetative tissues (Zhang and Brodribb 2017; Harrison Day et al. 2022), but it remains to be investigated whether this promotes flower retention during heat events.

Other reproductive injuries commonly observed following heat stress are anther indehiscence and poor pollen performance (Lohani et al. 2020). This occurs when heat coincides with pollination or the development of pollen and anthers (Hedhly et al. 2009). Because the anthers of many species remain hydraulically connected to the rest of the flower via the filament until anther dehiscence (Heslop-Harrison et al. 1987; Bonner and Dickinson 1990), or in some species until rapid filament extension just prior to anther dehiscence (Schmid 1976), declines in floral tissue water potential during heat in our study suggest that injuries of this nature could be in part driven by dehydration. Synchronous declines in corolla and anther water potential in tomato (*Solanum lycopersicum*) during heat support this notion (Bonner and Dickinson 1990). However, work on this topic has not explicitly separated the direct effect of temperature on pollen-related injuries from the indirect effect of localized floral dehydration.

Theoretical predictions and empirical data indicate that runaway cavitation in the flowering stem of mildly water stressed pyrethrum plants during transient heat induced a rapid decline in water potential resulting in the lethal desiccation of flowers. Validating the role of this process during heat-induced damage to flowers, and by extension, yield losses, and reproductive failure, highlights the importance of incorporating runaway cavitation into process-based modeling to understand the impact of hot and dry conditions on cultivated and natural plant systems. With rising global temperatures and changing rainfall patterns (IPCC 2014), obtaining a greater understanding of the impacts of combined heat and drought stress on plant reproduction is of the utmost urgency. The response of pyrethrum flower mortality to these stresses is likely to reflect a more general response of perennial plants to promote long-term survival of vegetative tissues; however, this remains to be tested. If commonalities exist, then the increasingly frequent co-occurrence of hot and dry weather with flowering (Hedhly et al. 2009) will have substantial negative impact on crop production, species’ persistence, and ecosystem function. We propose that the hydraulic and evaporative parameters found here to expose pyrethrum flowering stems to a greater risk of runaway cavitation than leaves during heat and drought stress provide a framework to examine the relative susceptibility of flowers in other plant species to hot and dry conditions.

## Materials and methods

### Plant material

Fourteen plants of the daisy pyrethrum (*T. cinerariifolium*) were sourced from a commercial growing site in northern Tasmania and established in 2-L pots filled with a mixture of 80% composted potting bark, 5% coarse potting sand, and 5% coco peat with slow-release fertilizer added. All plants were transferred to glasshouse facilities at the University of Tasmania where they experienced day and night temperatures of ∼21 and 18°C, respectively and ambient relative humidity. Plants received natural light, were watered to field capacity every day, and received weekly applications of liquid fertilizer (Peters Professional Winter Grow Special, Everris). Once plants were at least 5 mo old, they were vernalized for 3 wk in a growth cabinet with day and night temperatures of ∼20 and 6°C, respectively, a photosynthetic photon flux density (PPFD) of ∼750 *µ*mol m^−2^ s^−1^ and a photoperiod of 10 h to induce flowering (Brown and Menary 1994). Plants were then returned to initial conditions and produced flowers after ∼2 mo (Brown and Menary 1994).

### Residual transpiration and conductance to water vapor

The effect of temperature on residual transpiration (*E*_res_) was measured gravimetrically using detached flowers and leaves. Residual conductance to water vapor (*g*_res_) was subsequently calculated. Water was withheld from 3 pyrethrum plants until a predawn plant water potential of approximately −2.5 MPa was reached. It was assumed that this treatment would close stomata but not cause substantial embolism in the flowering stem. In pyrethrum stomatal conductance is reduced by 90% at a plant water potential of −2.1 MPa (Bourbia et al. 2021) and incipient embolism (*P*_12_) is triggered in the flowering stem at −3.2 MPa (Bourbia et al. 2020). Once the target water potential was reached, plants were transferred to the laboratory and kept in the dark overnight to ensure homogenous water potential among plant organs. Leaf water potential was measured with a Scholander pressure chamber (PMS Instrument Company) in the morning before plants were removed from the dark as a proxy for plant water potential prior to commencing measurements. Four mature flowers and 4 fully expanded leaves were excised from each plant to measure *E*_res_ at 2 temperatures: 19.4 ± 0.3 and 40.5 ± 0.2°C at a relative humidity of 55.5 ± 1.4 and 16.2 ± 0.4%, respectively (i.e. 2 replicates of each organ per temperature per plant). Measurements were performed in a controlled environment plant growth room. Organs were excised from plants, cut ends sealed with high-vacuum silicone grease (Dow Corning), and transferred to the growth room where they were weighed immediately and 10 min later using a ±0.0001 g analytical balance (MS204S, Mettler Toledo). It was assumed that organs had not dehydrated markedly beyond the water potential at excision during this time. A gentle flow of air was directed over the organs to disrupt the boundary layer using an air conditioner (FTXS50LVMA, Daikin Industries). Temperature and relative humidity were monitored with a temperature and relative humidity probe (EE181; Campbell Scientific) connected to a datalogger (CR10; Campbell Scientific). Leaf and flower samples included either the lamina or the flower head and ∼6 cm of the petiole or flowering stem. In between measurements, organs were positioned under a mixture of fluorescent and incandescent lights providing a PPFD of ∼500 *µ*mol m^−2^ s^−1^ on a wire mesh frame to raise them ∼1 cm above the bench surface. In 3 cases, 1 of the 2 replicates per plant–organ–temperature combination was discarded because a measurement was not completed within 10 min of excision. After the final measurement, an ∼1-mm-thick slice was removed from the cut end of flowers and leaves; cut ends were placed in water and left to rehydrate overnight. The following day flower heads were separated from flowering stems, and petals were removed from flower heads. The combined projected area of all dissected parts was then determined using a flatbed scanner (CanoScan CS8800F, Canon). *E*_res_ (mmol m^−2^ s^−1^) was normalized by the projected area of each organ (m^2^). *g*_res_ (mmol m^−2^ s^−1^) was subsequently calculated as:


(1) $$g_{\mathrm{res}}=\frac{E_{\mathrm{res}}\;\times P_{\mathrm{atm}}}{\mathrm{VPD}},$$

where *P*_atm_ is the atmospheric pressure (101.325 kPa) and VPD is the vapor pressure deficit calculated using the Buck equation (Buck 1981).


(2) $$\mathrm{VPD}\;=\;\;\left(1-\frac{\mathrm{RH}}{100}\right)\;\left(0.61121\;\;\times \;\;e^{\frac{17.502T}{240.97+T}}\right),$$

where *T* and RH are air temperature (°C) and relative humidity (%), respectively. Organ temperature was assumed to equal air temperature.

### Calculation to predict the point of runaway cavitation at the organ level

To understand the effect of contrasting leaf and flower water relations on organ susceptibility to runaway cavitation, we calculated the theoretical point of runaway cavitation for both at 20 and 40°C. To do this, we analytically derived a solution for the percentage loss of area-specific hydraulic conductance (*K*; mmol m^−2^ s^−1^ MPa^−1^) at runaway cavitation (PLC_runaway_; %) according to Tonet et al. (2023).

PLC_runaway_ was given by:


(3) $$\mathrm{PL}C_{\mathrm{runaway}}=\;\left(\frac{\frac{\alpha \;K_{\max}}{E_{\mathrm{res}}}}{1+\;\frac{\alpha \;K_{\max}}{E_{\mathrm{res}}}}\right),$$

where *K*_max_ is the maximum hydraulic conductance (mmol m^−2^ s^−1^ MPa^−1^) and *α* is a width parameter (MPa) that describes the relationship between increasing water stress and loss of hydraulic conductance due to cavitation.

*E*_res_ was calculated using the organ-specific *g*_res_ measured at 20 and 40°C in this study (Fig. 1) according to the following:


(4) $$E_{\mathrm{res}}=\;g_{\mathrm{res}}\;\left(\frac{VPD}{P_{\mathrm{atm}}}\right).$$

*E*_res_ was calculated assuming a temperature of 20 and 40°C, a relative humidity of 40% and 12%, and a VPD of 1.4 and 6.5 kPa, respectively. These conditions matched those experienced during plant growth and the heat treatment. Calculations of PLC_runaway_ used previously quantified *K*_max_ and *α* for pyrethrum (Bourbia et al. 2020). *K*_max_ and xylem vulnerability parameters were measured in this previous study using rehydration kinetics and the optical vulnerability technique, respectively (Bourbia et al. 2020). Hydraulic conductance was corrected in our calculations to account for the response of water viscosity to temperature using an empirical function based on data from Korson et al. (1969). Key input parameters are provided in Table 2.

**Table 2.** Key parameters used to calculate the theoretical loss of hydraulic conductance predicted to trigger runaway cavitation (PLC_runaway_)

		Flower		Leaf	
Parameter	Units	20°C	40°C	20°C	40°C
*K*_max_	mmol m^−2^ s^−1^ MPa^−1^	2.60	3.99	8.57	13.14
*P*_50_	MPa	3.57	3.57	6.48	6.48
*α*	MPa	0.2	0.2	0.65	0.65
*g*_res_	mmol m^−2^ s^−1^	12.15	13.98	4.68	5.01
*E*_res_	mmol m^−2^ s^−1^	0.17	0.9	0.06	0.32

Evaporative parameters were measured in this study and hydraulic parameters were taken from Bourbia et al. (2020).

### Simulations with the SurEau model

The mechanistic hydraulic model SurEau (Cochard et al. 2021) was used to simulate the impact of a short-term heat event on pyrethrum plants with the aim of predicting cavitation dynamics in flowering stems and leaves during a 180-min episode of high (40°C) temperature. SurEau simulates water flow and hydraulic pressure gradients based upon the principles of liquid flow through a porous system (the plant and soil), with detailed parameterization including internal and external hydraulic resistances in the plant and soil. In our simulations, SurEau calculated the flow of water from the soil to the atmosphere considering the hydraulic resistance (1/*K*) and the water storage capacity of 5 plant organs: the root, stem, branch, leaf, and flower. Water flow into flowers and leaves was driven by evaporative demand with each organ represented by a symplastic and apoplastic compartment, whose water dynamics in response to water potential depended on organ-specific pressure–volume curves and vulnerability curves, respectively. Water exchange between organs and compartments, hydraulic pressure, and resistance were computed at a very small time increment (0.001 s) whereas other processes including transpiration, cavitation, and the redistribution of water released by cavitation were computed at a longer time interval (60 s). After water flow was calculated, the water content and water potential of all organs were updated.

Seven separate simulations were performed in which a heat event commenced at different levels of drought stress (soil water potential = 0, −0.25, −0.5, −0.75, −1, −1.25, and −1.5 MPa). Parameter values were either taken from previous studies on the same species (Bourbia et al. 2020, 2021) or were estimated (details are provided in Supplemental Tables S2 and Data Set 1). All simulations were performed using a loam soil type (soil and climatic conditions are provided in Supplemental Table S2) assuming access to a finite soil water supply (soil volume: 0.054 m^3^ per 0.67 m^2^ combined leaf and flower projected area). Total fine root surface area was assumed to be equal to total leaf area. Total fine root length was 68.6 m. The ratio of total fine root length to soil volume was 1,275.2 m m^−3^. Leaf temperature was computed from the energy budget. Simulations accounted for the effect of temperature on water fluidity, surface tension, *P*_50_, and osmotic potential.

### Heat and drought treatment

**Figure 6.**
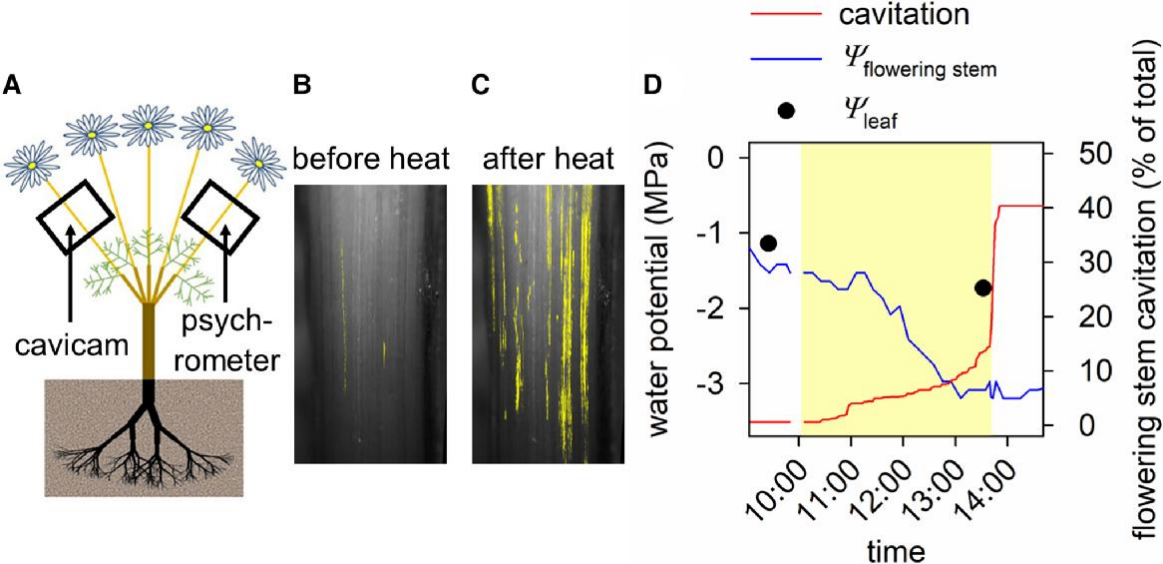
Flowering stem thickness and cavitation propagation monitored in situ before, during and after heat exposure. Flowering stem xylem was monitored for cavitation using cavicams fitted to plants in situ **A)**. Resulting image sequences reveal the cumulative xylem embolism and thickness of the flowering stem before **B)**, and after heat exposure **C)**. Yellow overlay on flowering stem images indicates the position of cumulative xylem embolisms. A stem psychrometer installed on a different flowering stem recorded changes to predawn water potential over time while water was withheld. The relationship between predawn water potential measured with the psychrometer and flowering stem thickness (% of maximum) was used to estimate flowering stem water potential. Together this data allowed us to estimate flowering stem water potential (*ψ*_flowering stem_) and monitor cavitation propagation during an experimental heat treatment (indicated by yellow shading) **D)**. Leaf water potential (*ψ*_leaf_) was also measured before and after heat exposure.

Having identified conditions likely to lead to runaway cavitation based on simulations in SurEau, we measured actual cavitation and water potential in flowering stems of potted pyrethrum plants exposed to a heat and drought treatment in situ and compared these with predicted xylem cavitation. In brief, plants were dehydrated to varying levels of water stress and then exposed to a short-term heat treatment, after which they were returned to growth conditions and rewatered. We selected 6 plants carrying 12 to 44 flowers between flower development stages 2 and 5 (Head 1966) with intact ray florets (petals) from those prepared as described above. Prior to heat exposure, plants were transferred to a glasshouse where day and night temperatures were regulated at 25 and 15°C, respectively. There plants experienced natural light and ambient relative humidity. A cavicam (Brodribb, Bienaimé, et al. 2016; Brodribb, Skelton, et al. 2016; Brodribb et al. 2017; Bourbia et al. 2020) (see http://www.opensourceov.org/ for detailed information regarding construction and use) was installed on 1 (or 2 in 1 case) flowering stem per plant to monitor cavitation propagation (for details see Cavitation monitoring section and Fig. 6) and flowering stem thickness. A stem psychrometer (ICT International) was also installed on a different flowering stem of each plant to measure predawn plant water potential. At the time of these measurements, evaporative demand was very low, and flowering stems were expected to have <50% cavitation. Thus, we assumed the hydraulic connection between flowering stem and the rest of the plant was sufficient to enable water potential equilibration. This allowed the individual relationship between flowering stem water potential and thickness to be established for each flowering stem and thus water potential estimated for the region of flowering stem viewed with the cavicam (for details, see Flowering stem water potential estimation section and Fig. 6). Water was then withheld from plants (or not withheld in 1 case) to apply drought stress ranging from a predawn plant water potential of 0 to −2.8 MPa. This range was selected to generate some embolism in the flowering stem of the most drought stressed plants but avoid complete hydraulic failure. Plants were then moved to the same controlled environment plant growth room described above in Residual transpiration and conductance to water vapor section, where the temperature was regulated at 40 ± 0.1°C and the relative humidity at 12.3 ± 1.3% resulting in a VPD of 6.5 ± 0.1 kPa. Lights provided a PPFD of ∼500 *µ*mol m^−2^ s^−1^. The well-watered plant was placed in a tray of water to maintain saturated soil water content. During the heat treatment (40°C for 3.5 h), flowering stem cavitation and water potential were monitored in situ. Leaf water potential was measured using a pressure chamber before plant exposure to heat and just before the heat treatment ended. After this, the temperature was reduced to 25°C, the lights switched off and plants left in the dark for 30 min. Leaf water potential was then measured again to estimate the plant water potential. Because evaporative demand was low when these measurements were made and leaves were expected to have no cavitation, we assumed the hydraulic connection between the leaf and the rest of the plant (excluding the flowering stems) was sufficient to enable water potential equilibration. Plants were subsequently returned to the glasshouse and rewatered to field capacity.

### Cavitation monitoring

Cavicams were installed on sentinel flowering stems to monitor cavitation using the optical vulnerability method (Brodribb, Bienaimé, et al. 2016; Brodribb, Skelton, et al. 2016; Brodribb et al. 2017; Bourbia et al. 2020). See http://www.opensourceov.org/ for detailed information regarding construction and use. One flowering stem was monitored on each of 5 plants and 2 on a sixth plant. A window (i.e. 1 side) of the epidermal tissue, cortex, and phloem, ∼15 mm in length and 4 mm in width, was carefully removed from 1 side of the flowering stem with a sharp razor to view the xylem (Bourbia et al. 2020). A layer of hydrogel (Tensive conductive adhesive gel, Parker Laboratories, Inc.) was applied to improve light transmission and reduce evaporation from the surface before the cavicam was secured in place. The cavicam was supported by a retort stand and clamp so that the flowering stem remained at its natural angle. Images from the flowering stem xylem were captured using reflected light every 2 min before and after heat exposure and every 1 min during heat exposure. The morning after heat exposure, each monitored flowering stem was excised at the base with the cavicam still attached and allowed to fully dehydrate in the dark under laboratory conditions (∼22°C and 60% relative humidity) until cavitation ceased and xylem was assumed to be 100% cavitated. Images were captured for at least 24 h after the last observed cavitation event. This allowed us to calculate the percentage of flowering stem xylem area that was cavitated before and after the heat treatment.

The resulting image sequences were analyzed to quantify the timing of cavitation propagation (Brodribb et al. 2017). Briefly, the rapid changes in light reflection which are observed when vessels transition from a water- to air-filled state during cavitation were quantified by determining the pixel difference between successive images. To do this, the pixel values of each image were subtracted from the next image in the sequence using ImageJ (National Institutes of Health) (see http://www.opensourceov.org/ for full details). Noise not associated with cavitation events was eliminated using the “remove outliers” function in ImageJ. The total embolism area per image was then calculated and expressed as a percentage of the total embolism area in the sequence (i.e. the cumulative embolism). Finally, cavitation propagation was plotted against time to determine the percentage of flowering stem vessels that were cavitated before and after heat exposure. Cavitation rate during heat exposure (% of total h^−1^) was also calculated as the change in cumulative embolism before and after heat divided by the time in hours over which the change occurred.

### Flowering stem water potential estimation

Psychrometry could not be used to monitor flowering stem water potential during the heat treatments because of its sensitivity to unstable thermal conditions. For this reason, flowering stem thickness was used as a proxy for water potential. Psychrometers were used to monitor flowering stem water potential during dehydration in the glasshouse before dawn when temperature fluctuations and water potential gradients within the plant were negligible. This permitted water potential to be estimated from the thickness of the region of flowering stem monitored for cavitation. The pyrethrum flowering stem is a determinate organ, meaning that changes in thickness are likely to be solely associated with changes in water potential, not growth. Thermal expansion of the flowering stem was considered negligible here because previous work found an average coefficient of thermal expansion in the radial direction of 1.34 × 10^−5^°C^−1^ for wet fresh wood of 5 tree species (Sevanto et al. 2005). Consequently, an increase in temperature from 25 to 40°C would cause an ∼0.02% increase in the thickness of a 4-mm-wide flowering stem. In contrast, flowering stem thickness decreased during heat by 1.24% to 7.74% in water stressed plants and increased by 0.95% in the well-watered plant.

**Figure 7.**
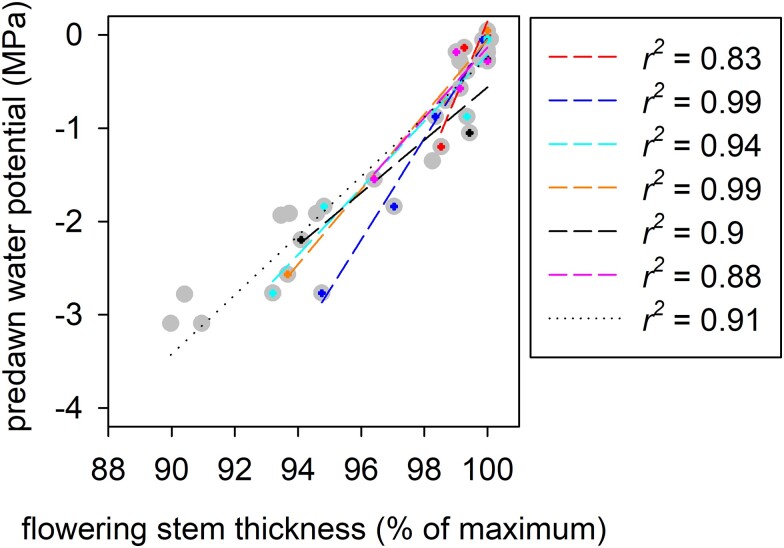
Flowering stems shrink in response to dehydration. Flowering stem thickness decreases in proportion to the predawn water potential when water is withheld from plants over several days. Different colored crosshair symbols indicate different flowering stems. Gray circles show pooled data comprising the 6 flowering stems shown individually in color and 3 additional flowering stems. Individual regressions were used to estimate flowering stem water potential (*Ψ*_flowering stem_) of those shown in color after heat exposure and a pooled regression (dotted line) was used to estimate flowering stem water potential of the well-watered plant after heat exposure [*Ψ*_flowering stem_ = 0.32 × flowering stem thickness (% of maximum)—32.03].

Thus, the relationship between flowering stem predawn water potential and thickness during plant dehydration prior to the heat treatment was used to estimate flowering stem water potential before and after heat exposure. To do this, the relationship between predawn water potential and flowering stem thickness was established for each flowering stem (except the flowering stem from the well-watered plant). Flowering stem thickness was derived from images captured to monitor cavitation and expressed as a percentage of the maximum when plants were hydrated to field capacity. Thickness was measured using ImageJ either with the “threshold” function if there was enough contrast between tissue and background or the line tool if not, and a linear regression was fitted for each flowering stem (Fig. 7). Relationships from the 6 drought-stressed flowering stems (from 5 plants; 2 flowering stems were on the same individual) were pooled and supplemented with data from 3 additional drought-stressed flowering stems (from 2 plants; 1 flowering stem from 1 plant and 2 from a second plant) to estimate the flowering stem water potential of the well-watered plant before and after heat exposure (Fig. 7).

### Flower mortality

Mortality of individual flower heads downstream (in the direction of the transpiration stream) from each flowering stem monitored for cavitation was scored based on ray floret (petal) turgor the day after heat exposure and rewatering. Because pyrethrum flowers are quite long-lived with petals remaining turgid for 26 to 30 d under nonstressful glasshouse conditions (Bourbia et al. 2020) and ∼31 d in the field (Head 1966), we assumed that loss of petal turgor scored immediately after heat was heat related and not due to natural flower senescence. Loss of petal turgor was previously linked with reduction in the floral disc expansion (Bourbia et al. 2020) normally associated with achene development (Suraweera et al. 2017). Thus, flowers were considered to have died when petals remained wilted the day after the heat treatment. In some cases, only a subset of petals within individual flowers lost turgor or only the distal tips of petals were dehydrated. These flowers were scored as dead if >50% of petals within a flower lost turgor and/or if petals with dehydrated tips also lost turgor. Mortality of the entire flower canopy was then scored for each plant in the same way and expressed as a percentage of total flowers per plant. Floral bud diameter was also measured using a digital calliper with a resolution of ±0.01 mm (IP54, Moore and Wright) the day after heat exposure and rewatering, and again a week later. Buds were scored as dead if they failed to expand during this time. Mortality of the entire bud canopy was expressed as a percentage of total buds per plant.

Flower canopy mortality was scored in the same way for 3 additional plants exposed to water stress under mild temperatures. These plants remained in the initial mild environmental conditions described above in Plant material section. The petals of all flowers were healthy and showed no signs of wilting before water was withheld from plants. Water was withheld until a flowering stem water potential known to generate cavitation in >55% of flowering stem xylem area was reached (range: 67.5% to 99.2%). Water potential was measured daily at midday with a Scholander pressure chamber using leaves attached to the flowering stem that had been enclosed in plastic wrap and aluminum foil for at least 30 min. Under these circumstances, leaf water potential was assumed to be in equilibrium with flowering stem water potential. Once the target water potential was reached, plants were rewatered. Petal condition was assessed the next day.

### Leaf damage

The effect of heat exposure on leaves was assessed by comparing the chlorophyll fluorescence ratio Fv/Fm (a proxy for photosynthetic damage) before and after the heat treatment. Measurements were made on 10 leaves per plant that had been dark adapted for 1 h a few days before water was withheld, and plants underwent the heat treatment using a Portable Chlorophyll Fluorometer (PAM-2000, Walz). Measurements were repeated on the same 10 leaves per plant a few days after the heat treatment.

### Data analysis

Two-way ANOVA was performed in R (R Core Team 2017) to test the effect of organ type and temperature on residual conductance to water vapor and transpiration. Data were log and square root transformed, respectively, to ensure homoscedasticity and normality. A 2-tailed paired student's *t*-test was used to test whether plant water potential or chlorophyll fluorescence parameter Fv/Fm in leaves varied before and after exposure to heat and whether the magnitude of decline in water potential before and after heat exposure varied among flowering stems and leaves. The water potential at which air entered the flowering stem and leaf xylem (*P*_12_) was determined by fitting a sigmoid function to the relationship between cavitation (% of total) and water potential (Bourbia et al. 2020) using the following equation (Pammenter and Van der Willigen 1998):


(5) $$\mathrm{Cumulative}\;\mathrm{cavitation}\;\;=\;\;\frac{100}{1+\;e^{\left(a(\Psi -P_{50})\right)}},$$

where *a* is a fitted parameter related to the slope of the curve, *ψ* is the flowering stem water potential expressed as a negative value (−MPa), and *P*_50_ is the flowering stem water potential at which 50% of the cavitation events had been observed (−MPa). This equation was then rearranged to:


(6) $$\Psi =\left(\frac{\ln\;\left(\left(\frac{100}{\mathrm{cumulative}\;\mathrm{cavitation}}\right)-1\right)}{a}\right)+\;P_{50},$$

to calculate *P*_12_; the flowering stem water potential at which 12% of the cavitation events had been observed.

## Supplementary Material

kiad349_Supplementary_Data

## Data Availability

Raw image data are available from the corresponding author upon reasonable request.

## Supplemental data

The following materials are available in the online version of this article.

**Supplemental Figure S1.** Cavitation propagation rate during experimental heat stress increased with existing cavitation load in flowering stems before heat.

**Supplemental Figure S2.** Plant water potential before and after experimental heat stress.

**Supplemental Figure S3.** Mature flowers were more sensitive to experimental heat stress than developing flower buds.

**Supplemental Table S1.** Flower canopy mortality (% of total) following drought but no heat stress.

**Supplemental Table S2.** Main parameters used in simulations with the model SurEau to predict cavitation dynamics in flowers and leaves during a short-term episode of high (40°C) temperature.

**Supplemental Data Set 1.** MS excel file with the parameters and climatic conditions for all simulations.
