# Supplementary material for: Combined heat and water stress leads to local xylem failure and tissue damage in pyrethrum flowers
Source: Plant Physiol. 2023 Jun 16;193(1):356–70. doi: 10.1093/plphys/kiad349 (PMC10469517; doi:10.1093/plphys/kiad349)
Supplement: kiad349_Supplementary_Data [file kiad349_supplementary_data.zip › Supplemental_Data.pdf]

This file includes:

Supplemental Figures S1 to S3

Supplemental Tables S1 to S2

Legend for Supplemental Dataset S1

SI References

Other supplementary materials for this manuscript include the following:

Supplemental Dataset S1

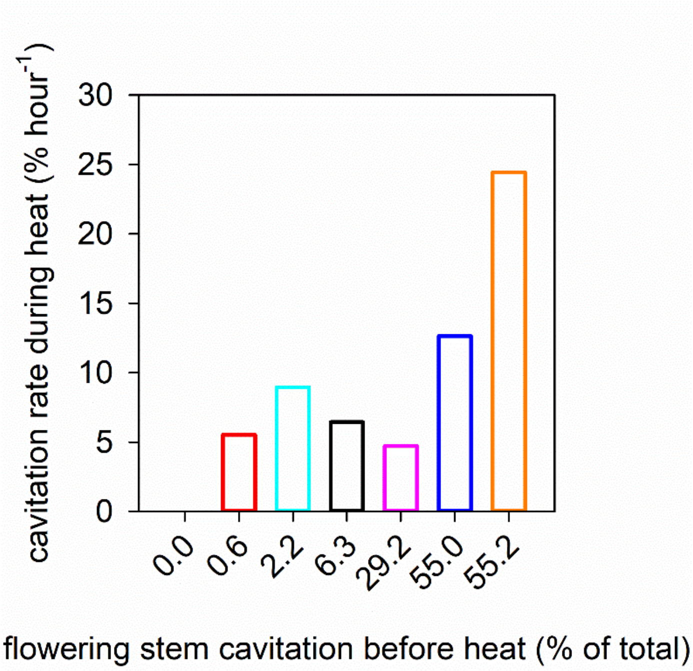

Supplemental Figure S1. Cavitation propagation rate during experimental heat stress increased with existing cavitation load in flowering stems before heat. Relationship between flowering stem cavitation (% of total) before heat exposure (cavitation load) and the rate of cavitation propagation. Different colors represent different flowering stems.

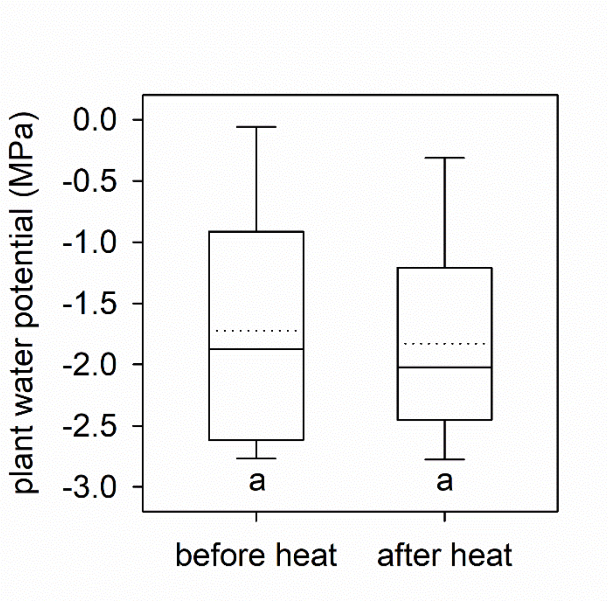

Supplemental Figure S2. Plant water potential before and after experimental heat stress. Box plot whiskers show data range, top of each box shows the upper quartile, dotted lines show means, solid lines show medians, and the bottom of each box shows the lower quartile (n = 6 individuals). Same letter indicates no significant difference ($P > 0.05$) by two-tailed paired student's t-test.

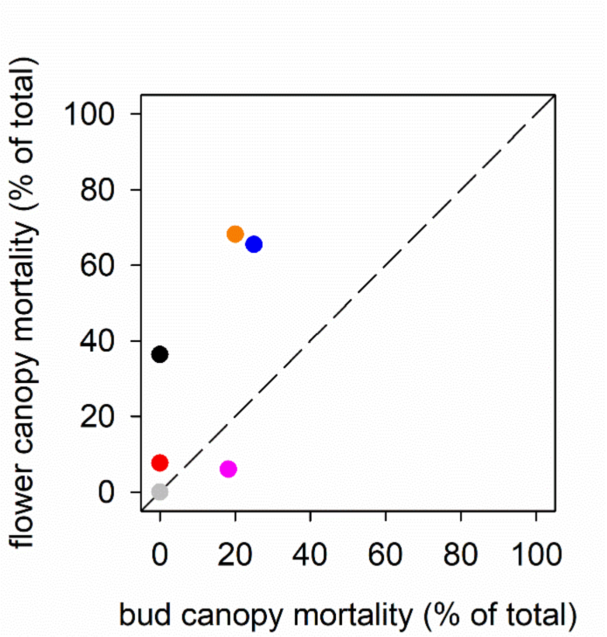

Supplemental Figure S3. Mature flowers were more sensitive to experimental heat stress than developing flower buds. Flower canopy mortality (% of total mature flowers per plant) versus bud canopy mortality (% of total buds per plant). Dashed line shows the 1:1 relationship. Different color symbols represent values from different individuals.

Supplemental Table S1. Flower canopy mortality (% of total) following drought but no heat stress.

**Lowest flowering stem water potential (MPa)**	**Predicted loss of flowering stem conductance (%)**	**Canopy flower mortality (% of total)**
-3.72	67.5	0
-4.01	83.2	0
-4.6	99.2	33

Supplemental Table S2. Main parameters used in simulations with the model SurEau to predict cavitation dynamics in flowers and leaves during a short-term episode of high (40 °C) temperature.

SurEau inputs	Name	Units	Value	Source
	time increment	s	0.001	
**climate**	air temperature	°C	25	
	air temperature during heat event	°C	40	
	relative humidity	%	30	
	photosynthetically active radiation	µmol	1200	
	wind speed	m s^{-1}	1	
**soil**	total saturated water content for loam	unitless	0.399	(Van Genuchten, 1980; U.S. Department of Agriculture, 2019)
	residual water content for loam	unitless	0.061	(Van Genuchten, 1980; U.S. Department of Agriculture, 2019)
	Van Genuchten parameter related to soil air entry water potential for loam	cm^{-1}	0.0111	(Van Genuchten, 1980; U.S. Department of Agriculture, 2019)

	Van Genuchten parameter related to the pore size distribution in soil particles for loam	unitless	1.472	(Van Genuchten, 1980; U.S. Department of Agriculture, 2019)
	hydraulic conductivity at saturation for loam	mol s^{-1} MPa^{-1}	12.70	(Van Genuchten, 1980; U.S. Department of Agriculture, 2019)
	Van Genuchten parameter	unitless	0.5	(Van Genuchten, 1980)
	soil volume	m^3	0.054	
**leaf stomata/cuticle**	total plant leaf area	m^2	0.2155	(Bourbia et al., 2020)
	leaf symplasmic water potential causing 12 % of stomatal closure	MPa	-1.5	(Bourbia et al., 2020)
	leaf symplasmic water potential causing 88 % of stomatal closure	MPa	-3.03	(Bourbia et al., 2020)
	maximum stomatal conductance	mmol s^{-1} m^{-2}	273.9	(Bourbia et al., 2020)
	minimum residual leaf conductance	mmol s^{-1} m^{-2}	4.682	this study
**flower stomata/cuticle**	total plant flower area	m^2	0.4516	estimated from flower projected area

	flower symplasmic water potential causing 12 % of stomatal closure	MPa	-1.5	assumed to be the same as the leaf
	flower symplasmic water potential causing 88 % of stomatal closure	MPa	-3.03	assumed to be the same as the leaf
	maximum flower diffusive conductance	mmol s^{-1} m^{-2}	67.39	(Bourbia et al., 2020)
	minimum residual flower conductance	mmol s^{-1} m^{-2}	13.066	this study
**leaf hydraulics**	leaf xylem P50	MPa	-6.48	(Bourbia et al., 2020)
	slope of the linear portion of leaf vulnerability curve	% MPa^{-1}	38.25	(Bourbia et al., 2020)
	leaf apoplasmic conductance per unit area	mmol s^{-1} MPa^{-1} m^{-2}	39	(Bourbia et al., 2020)
	leaf symplasmic conductance per unit area	mmol s^{-1} MPa^{-1} m^{-2}	11	(Bourbia et al., 2020)
	leaf apoplasmic capacitance per unit area	kg MPa^{-1} L^{-1}	0.0002	estimated from whole plant capacitance given in (Bourbia et al., 2021)
	leaf modulus of elasticity	MPa	10	estimated
	leaf osmotic potential at full turgor	MPa	-1.5	estimated
	leaf water content per unit area	g m^{-2}	100	estimated
**flower hydraulics**	peduncle xylem P50	MPa	-3.57	(Bourbia et al., 2020)

	slope of the linear portion of peduncle vulnerability curve	% MPa^{-1}	122.2	(Bourbia et al., 2020)
	apoplasmic conductance per floral organ	mmol s^{-1} MPa^{-1}	2.0944	calculated assuming xylem area specific conductivity of the flowering stem is the same as the branch
	symplasmic conductance between peduncle and flower symplasm per floral organ	mmol s^{-1} MPa^{-1}	0.00512	(Bourbia et al., 2020)
	symplasmic conductance between peduncle apoplasm and peduncle symplasm per floral organ	mmol s^{-1} MPa^{-1}	0.0188	calculated assuming xylem area specific conductivity of the flowering stem is the same as the branch
	flower apoplasmic capacitance per unit area	kg MPa^{-1} L^{-1}	0.002	estimated from whole plant capacitance given in (Bourbia et al., 2021)
	flower modulus of elasticity	MPa	7.55	estimated
	flower osmotic potential at full turgor	MPa	-1.25	estimated
**root hydraulics**	root xylem P50	MPa	-6.48	assumed to be the same as the leaf

slope of the linear portion of root vulnerability curve	% MPa^{-1}	38.25	assumed to be the same as the leaf
terminal root sapwood area specific conductivity	mmol m s^{-1} MPa^{-1} m^{-2}	10000	estimated
root surface area symplasmic specific conductance	mmol s^{-1} MPa^{-1} m^{-2}	10	estimated
root apoplasmic capacitance per unit area	kg MPa^{-1} L^{-1}	0.002	estimated from whole plant capacitance given in (Bourbia et al., 2021)
root modulus of elasticity	MPa	10	estimated
root osmotic potential at full turgor	MPa	-1.5	estimated
coefficient of power function describing the relationship between root symplasmic conductance and soil water potential	unitless	1.2214	(Bourbia et al., 2021)
exponent of power function describing the relationship between root symplasmic conductance and soil water potential	unitless	-1.97	(Bourbia et al., 2021)

Supplemental Dataset S1 (separate file). MS excel file with the parameters and climatic conditions for all simulations.

SI References

Bourbia I, Carins-Murphy MR, Gracie AJ, Brodribb TJ (2020) Xylem cavitation isolates leaky flowers during water stress in pyrethrum. New Phytologist **227:** 146-155

Bourbia I, Pritzkow C, Brodribb TJ (2021) Herb and conifer roots show similar high sensitivity to water deficit. Plant Physiology **186:** 1908-1918

U.S. Department of Agriculture (2019) ROSETTA Class Average Hydraulic Parameters. Available at: https://www.ars.usda.gov/pacific-west-area/riverside-ca/agricultural-water-efficiency-and-salinity-research-unit/docs/model/rosetta-class-average-hydraulic-parameters/ (Accessed: 29th June 2022).

Van Genuchten MT (1980) A closed-form equation for predicting the hydraulic conductivity of unsaturated soils. Soil Science Society of America Journal **44:** 892-898
